# Efficacy and safety of catheter ablation as first-line therapy for the management of ventricular tachycardia

**DOI:** 10.1007/s10840-023-01483-2

**Published:** 2023-02-09

**Authors:** Yasuhito Kotake, Kaimin Huang, Richard Bennett, Kasun De Silva, Ashwin Bhaskaran, Juliana Kanawati, Samual Turnbull, Julia Zhou, Timothy Campbell, Saurabh Kumar

**Affiliations:** 1 Department of Cardiology, Westmead Hospital, Sydney, NSW Australia; 2 Westmead Applied Research Centre, University of Sydney, Corner Hawkesbury and Darcy Roads, Westmead, Sydney, NSW 2145 Australia

**Keywords:** Implantable cardioverter defibrillation, Anti-arrhythmic drug, Catheter ablation, Ventricular tachycardia, First-line therapy

## Abstract

**Background:**

Ventricular tachycardia (VT) is associated with significantly increased morbidity and mortality. Catheter ablation (CA) in line with an implantable cardioverter-defibrillator (ICD) is highly effective in VT management; however, it is unknown if CA should be considered as first-line therapy. The aim of this study is to verify the efficacy and safety of CA as first-line therapy for the first VT presentation (as adjunctive to ICD insertion), compared to initial ICD insertion and anti-arrhythmic drug (AAD) therapy.

**Methods:**

Data from patients with the first presentation for VT from January 2017 to January 2021 was reviewed. Patients were classified as “ablation first” vs “ICD first” groups and compared the clinical outcomes between groups.

**Results:**

One hundred and eighty-four consecutive patients presented with VT; 34 underwent CA as first-line therapy prior to ICD insertion, and 150 had ICD insertion/AAD therapy as first‐line. During the median follow-up of 625 days, patients who underwent CA as first-line therapy had significantly higher ventricular arrhythmia (VA)-free survival (91% vs 59%, log-rank *P* = 0.002) and composite of VA recurrence, cardiovascular hospitalization, transplant, and death (84% vs 54%, log-rank *P* = 0.01) compared to those who did not undergo CA. Multivariate analysis revealed that first-line CA was the only protective predictor of VA recurrence (hazard ratio (HR) 0.20, *P* = 0.003). There were 3 (9%) peri-procedural complications with no peri-procedural deaths.

**Conclusion:**

Real-world data supports the efficacy and safety of CA as first-line therapy at the time of the first VT hospitalization, compared to the initial ICD implant and AAD therapy.

**Supplementary Information:**

The online version contains supplementary material available at 10.1007/s10840-023-01483-2.

## Introduction

Ventricular tachycardia (VT) is a major cause of sudden cardiac death. Implantable cardioverter-defibrillators (ICD) provide a mortality benefit and prevent sudden cardiac death prevention in patients with VT [1]. However, the presence of an ICD itself does not reduce the risk of VT onset nor does it reduce VT burden. Regardless of underlying etiology, patients with a secondary prevention ICD experience a higher incidence of subsequent ICD shocks from recurrent VT, than those with a primary prevention indication [2, 3]. ICD shocks are painful, reduce the quality of life, and portend an increased risk of heart failure and mortality [4, 5]. Therefore, it is reasonable to surmise that every effort should be made to minimize the risk of subsequent VT recurrence which activates ICD shock. Catheter ablation (CA) is highly effective for VT management, but is often reserved as a treatment of last resort [6–9].

Retrospective studies have shown that delayed referral for ablation, when one or more ICD shocks, and/or anti-arrhythmic drug (AAD) failures have occurred, often results in a poorer prognosis [10–12]. BERLIN-VT was a randomized trial that evaluated the optimal timing of VT ablation, showing that early ablation was associated with reduced incidence of sustained ventricular arrhythmia (VA) and appropriate ICD therapies; however, results were of borderline statistical significance [13]. Recently, PARTITA showed that patients randomized to ablation, compared to medical therapy after the first ICD shock, experienced fewer ICD shocks in follow-up, as well as a lower incidence of a composite endpoint of all-cause mortality and hospitalization for heart failure in follow-up [9]. Furthermore, PAUSE-SCD showed that at the time of first presentation for VT, patients randomized to upfront catheter ablation with concurrent ICD implantation, compared to medical therapy, experienced a lower composite primary outcome of VT recurrence, cardiovascular hospitalization, or death, driven mainly by a reduction in ICD therapies [14].

To extend the findings of the randomized trials PARTITA and PAUSE-SCD to the real-world setting, we hypothesized that VT ablation at the time of the first presentation with VT prior to concurrent ICD insertion would be more efficacious than initial medical therapy and ICD insertion and evaluated this in a retrospective analysis.

## Methods

### Study participants

**Fig. 1:**
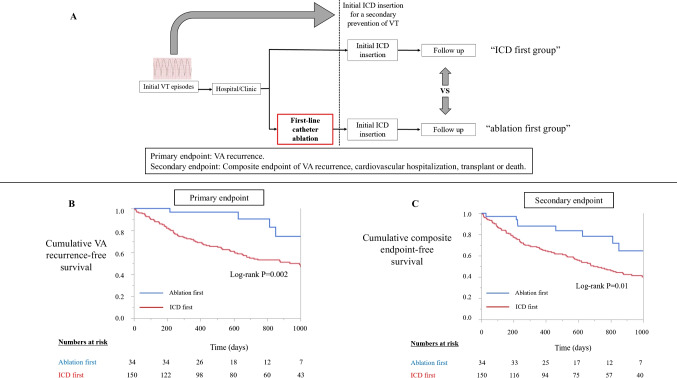
**A** Study design. During the study period, patients presenting with sustained monomorphic VT requiring a secondary prevention ICD were reviewed. Patients were classified into “ablation first” or “ICD first” groups, based on clinician preference. **B** Survival-free from recurrent VA after ICD insertion. Event-free survival analysis for the VA recurrence showed that patients in ICD first group had a significantly higher cumulative incidence for the VA recurrence than patients in the ablation first group (log-rank test; *P* = 0.002). **C** Overall survival-free from the composite endpoints of VA recurrence, cardiovascular hospitalization, transplant, and death after ICD insertion. Event-free survival analysis for the composite endpoints VA recurrence, cardiovascular hospitalization, transplant, and death after discharge showed that patients in the ICD first group had a significantly higher cumulative incidence for the composite endpoints than patients in the ablation first group (log-rank test; *P* = 0.01)

Between January 2017 and January 2021, prospectively collected registry data from the Westmead Hospital Ventricular Arrhythmia registry on patients presenting with sustained monomorphic VT requiring a secondary prevention ICD at Westmead Hospital, Sydney, Australia, were reviewed (Fig. 1A). Primary prevention ICD recipients were excluded. All patients underwent echocardiography and/or cardiac magnetic resonance (CMR) imaging to screen for the presence of structural heart disease and to define the ventricular function before ICD insertion. The distinction between ischemic cardiomyopathy (ICM) and non-ischemic cardiomyopathy (NICM) was based primarily on the presence of relevant coronary artery disease, which was confirmed with coronary angiography. NICM was identified as an absence of relevant coronary artery disease and defined according to the criteria of the ESC Working Group on Myocardial and Pericardial Diseases [15]. Written informed consent was obtained in all cases as part of routine clinical care. An analysis of this data was approved by the Human Research Ethics Committee of Westmead Hospital.

#### Study groups

Patients were defined as “ablation first” vs “ICD first” groups, based on clinician preference. There was no randomization.

- “ablation first” group: this group received a first-line CA, which was defined as VT ablation performed at the time of the first VT hospitalization, prior to concurrent ICD insertion. Patients who had already administered AADs as a result of emergency for VT management prior to CA were also included if VT ablation was performed at the time of the first VT hospitalization, prior to concurrent ICD insertion.
- “ICD first” group*:* this group received AADs and concurrent ICD implant, without CA at the time of the first VT hospitalization.

### Ventricular tachycardia storm

VT storm was defined as (i) ≥ 3 separate episodes in 24 h (h), each requiring termination by intervention; (ii) frequent defibrillator therapies (≥ 3 separate episodes separated by 5 min in 24 h); or (iii) incessant ventricular tachycardia (continuous VT that recurred promptly despite intervention for termination over 12 h) [16].

### Catheter ablation procedures

VT was induced utilizing an induction protocol, as described previously [17]. Programmed electrical stimulation (PES) was performed from at least 2 right ventricular (RV) sites using a 400-ms drive train with 4 extra-stimuli beginning at 300 ms, decrementing by 10 ms down to ventricular refractoriness. LV stimulation was used if VT was non-inducible after RV stimulation. This was followed by burst RV pacing down to ventricular refractoriness from the RV apex. PES and burst RV pacing were then repeated from each site using the highest tolerated dose of isoprenaline with hemodynamic support to maintain perfusion pressure with inotropic or mechanical circulatory support initiated at the start of the case. Isoprenaline was initially commenced at 10 µg/min, with the PES and RV burst pacing protocol repeated after incrementing the isoprenaline dose by 10 µg/min up to 40 µg/min. Sustained VT was defined as monomorphic ventricular arrhythmia with a duration > 10 s. All induced VTs were targeted for ablation.

Ablation was performed using an irrigated catheter (Navistar Smarttouch Thermocool™ or Navistar Smarttouch Thermocool SF™, Biosense Webster). The endpoint of each ablation lesion was controlled with ≥ 10 g (g) contact force (CF) aiming for an impedance drop of ≥ 20 ohms and a maximum power of 50 watts (W), applied in a power-controlled mode. Where possible, ablation lesions were repeated until the site was electrically unexcitable with pacing at 10 mA at 9 ms pulse width. Ablation was guided by substrate mapping and/or activation mapping. Ablation targeted presumptive isthmus and exit, based on activation and entrainment mapping, if the VT was hemodynamically tolerated. If the VT was not tolerated or short in duration, a substrate-based ablation was performed for scar-related VTs [18].

### Acute procedural outcomes

Acute procedural outcomes were classified as complete success (non-inducibility of any VTs), partial success (elimination of at least 1 VT), and failure (residual inducibility of clinical/spontaneous VT) [19]. Patients with only non-sustained VT [20] or fast VTs [21] at the end of the procedure were defined as non-specific VTs and classified as non-inducible VT.

### ICD programming

Two VT treatment zones were programmed. The first zone was programmed below the rate of the slowest VT (with/without or anti-tachycardia pacing (ATP)), and the second zone was programmed at a minimum detection rate of > 188 beats per minute and programmed to deliver a shock (with/without ATP).

### Outcomes

The primary endpoint assessed was freedom from VA recurrence. The secondary endpoint was a composite of survival free of VA recurrence, cardiovascular hospitalization, transplant, or death.

### Follow-up

All patients were enrolled in a remote monitoring service, managed either by Westmead Hospital or the referring cardiologist. All ICD activations were recorded and transmitted to the clinic, which prompted an in-office visit for a detailed evaluation of the clinical and device data. The hospital medical records and outpatient clinic assessments were used to complete the clinical follow-up. The recurrence of VA was defined based on existing clinical guidelines as VT lasting greater than 30 s or sustained VT or ventricular fibrillation (VF) requiring ICD therapies (shocks or ATP). Inappropriate ICD therapy was excluded from VA recurrence. Follow-up was defined from the date of the CA (ablation first group) or ICD insertion (ICD first group) to the last clinical follow-up,

### Statistical analysis

Continuous variables were expressed as the mean ± standard deviation (SD) if normally distributed: median and 25–75% interquartile range (IQR) or full ranges were used if the data were clearly skewed. Categorial variables were expressed as frequencies and percentages. Continuous variables were compared using Student’s *t*-test when normally distributed or a Mann–Whitney *U* test when they were not normally distributed. A chi-squared test was used when comparing categorical variables or Fisher’s exact test when required. The estimated event-free survival probabilities were calculated using a Kaplan–Meier method, and log-rank tests were used for group comparisons. Cox proportional hazard models were created to determine predictors of VA recurrence. Hazard ratios (HR) and 95% confidence intervals (CI) were used to express the risk of VT recurrence. A two-tailed *P*-value < 0.05 was considered statistically significant. All statistical analyses were performed with JMP version 14 software (SAS Institute Inc., Cary, NC, USA).

## Results

### Study Participants

**Fig. 2:**
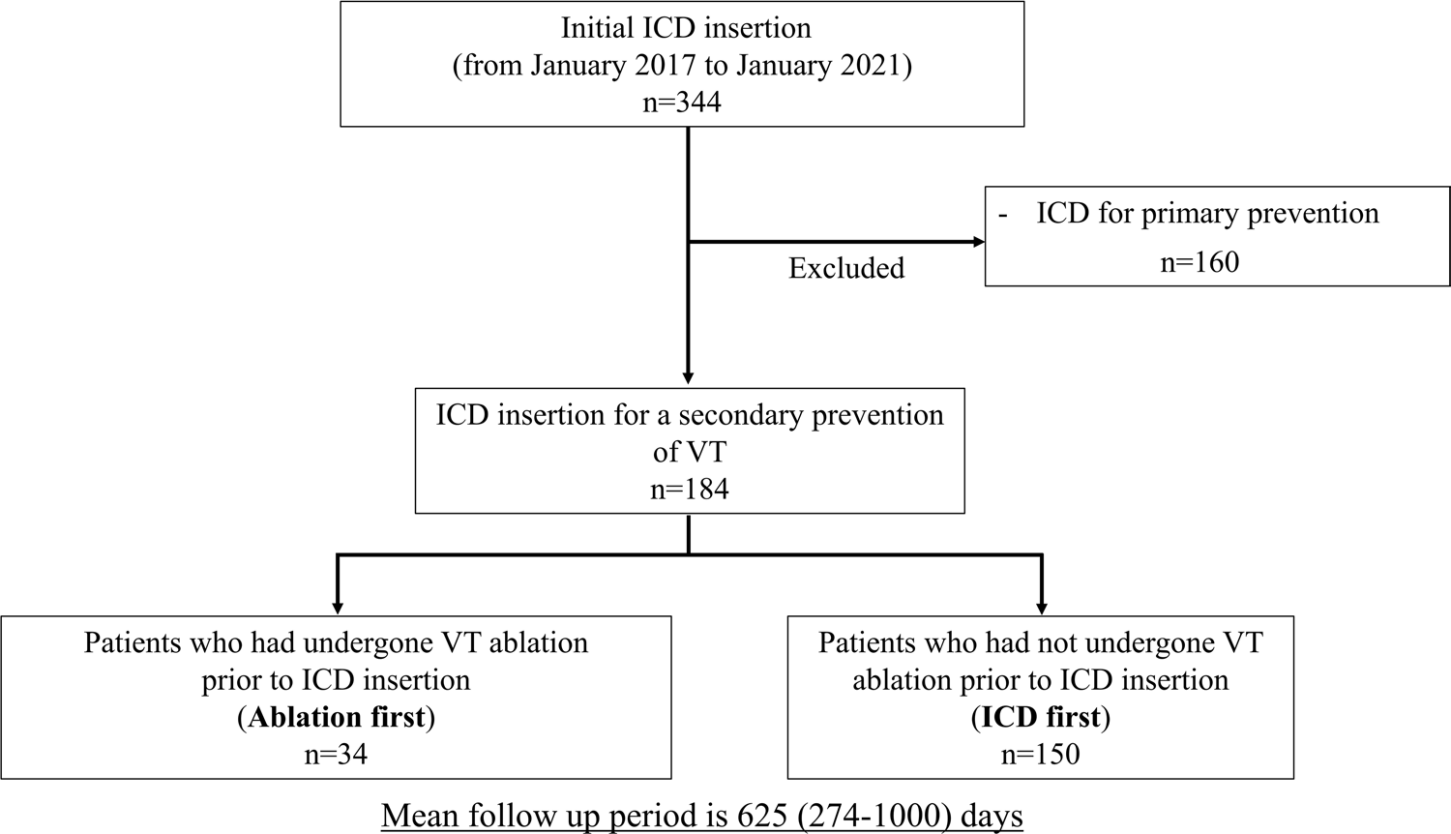
Study enrolment. During study periods, 344 patients who underwent initial ICD insertion in Westmead hospital were reviewed. One hundred-sixty patients were excluded since their indication was for primary prevention purposes. As a result, 184 patients were included in this study. Thirty-four patients were in the “ablation first group,” and 150 patients were in the “ICD first group”

During the study period, 344 patients who underwent initial ICD insertion in Westmead hospital were reviewed (Fig. 2). Of these, 160 patients were excluded since they underwent ICD implantation for primary prevention. As a result, 184 patients were included in this study. Thirty-four patients were in the “ablation first group,” and 150 patients were in the “ICD first group.”

### Baseline characteristics

Baseline characteristics are presented in Table 1. Baseline characteristics were comparable between the two groups except for left ventricular ejection fraction (LVEF) and storm presentation. The NICM etiology of underlying heart disease was present in 54% of patients. Patients in the ablation first group showed marginally higher LVEF than the ICD first group (45% (IQR: 40–51.5) vs 41% (IQR: 33–49), *P* = 0.04). Storm presentation was more prevalent among patients in the ablation first group compared with the ICD first group (35% vs 5%, *P* = 0.002). In terms of the prevalence of atrioventricular or intraventricular conduction block, there were no significant differences between groups. QTc interval was slightly prolonged in ICD first group, but there was no significant difference in both groups (434 (411–465) ms vs 454 (421–480) ms, *P* = 0.17). Class III AADs at the time of the first VT admission tended to be used more often in the ICD first group, but there was no statistically significant difference between the two groups (ablation first group vs ICD first group: 32% vs 43%, *P* = 0.26). Guideline-driven therapy for heart failures such as beta-blockers, angiotensin-converting enzyme (ACE) inhibitors/angiotensin receptor blockers (ARBs), mineralocorticoid receptor antagonist (MRA), sodium-glucose cotransporter-2 (SGLT2) inhibitor, and angiotensin receptor-neprilysin inhibitor (ARNI) was equally prescribed for both groups.

**Table 1:** Baseline characteristics

	Ablation first (*n* = 34)	ICD first (*n* = 150)	*P* value
Baseline characteristics			
Age (y)	59 ± 18	61 ± 15	0.49
Male gender (*n*, %)	25 (74)	115 (77)	0.66
LVEF (%)	45 [40–51.5]	41 [33–49]	0.04*
BMI	29.5 (26.0–34.2)	29.8 (25.5–33.8)	0.39
eGFR	70 ± 20	73 ± 21	0.51
Aetiology of heart disease			
ICM (*n*, %)	13 (38)	72 (48)	0.34
NICM (*n*, %)	21 (62)	78 (52)	0.34
Comorbidities			
Hypertension (*n*, %)	15 (44)	88 (59)	0.13
Diabetes mellitus (*n*, %)	9 (26)	49 (33)	0.54
Dyslipidemia (*n*, %)	17 (50)	93 (62)	0.25
Smoking (*n*, %)	5 (15)	32 (21)	0.48
Atrial fibrillation (*n*, %)	9 (26)	55 (37)	0.32
COPD (*n*, %)	2 (6)	13 (9)	0.74
Device type on discharge			
ICD (*n*, %)	27 (79)	122 (81)	0.81
CRT-D (*n*, %)	7 (21)	28 (19)	0.81
Physical status on admission (at the time of first VT admission)			
Heart failure (*n*, %)	5 (15)	28 (19)	0.80
Storm presentation (*n*, %)	12 (35)	8 (5)	0.002*
Electrocardiography characteristics at baseline			
Atrio-ventricular conduction block			
IAVB (*n*, %)	6 (18)	29 (19)	1.00
CAVB (*n*, %)	5 (15)	16 (11)	0.55
Intraventricular block			
RBBB (*n*, %)	2 (6)	16 (11)	0.53
LBBB (*n*, %)	3 (9)	14 (9)	1.00
LAFB (*n*, %)	1 (3)	6 (4)	1.00
RBBB + LAFB (*n*, %)	1 (3)	0 (0)	0.18
Electrocardiography parameters			
QRS width (ms)	112 [93–125]	110 [98–148]	0.16
QTc interval (ms)	434 [411–465]	454 [421–480]	0.17
Class III anti-arrhythmic drugs (at the time of admission/pre ablation)			
Class III anti-arrhythmic drugs (*n*, %)	11 (32)	65 (43)	0.26
Amiodarone (*n*, %)	5 (15)	40 (27)	0.19
Sotalol (*n*, %)	6 (18)	25 (17)	1.00
Class III anti-arrhythmic drugs (at the time of discharge/post ablation)			
Class III anti-arrhythmic drugs (*n*, %)	6 (18)	65 (43)	0.006*
Amiodarone (*n*, %)	3 (9)	40 (27)	0.03*
Sotalol (*n*, %)	3 (9)	25 (17)	0.30
Guideline driven-therapy for heart failure (at the time of first VT admission)			
Beta-blocker (*n*, %)	24 (71)	117 (78)	0.37
ACE inhibitor/ARB (*n*, %)	17 (50)	81 (54)	0.71
MRA (*n*, %)	4 (12)	32 (27)	0.07
SGLT2 inhibitor (*n*, %)	3 (9)	8 (7)	0.71
ARNI (*n*, %)	4 (12)	16 (13)	1.00
Coronary intervention			
PCI (*n*, %)	8 (24)	35 (23)	1.00
CABG (*n*, %)	6 (18)	29 (19)	1.00

^*^Continuous variables compared using a Student *t*-test when normally distributed, or a Mann–Whitney *U* test when they were not normally distributed. Chi-squared test was used when comparing categorical variables or Fisher’s exact test when required. *P* values were considered statistically significant when 0.05 or less

Abbreviations: *ACE*, angiotensin-converting enzyme; *ARB*, angiotensin receptor blockers; *ARNI*, angiotensin receptor-neprilysin inhibitor; *AVB*, atrio-ventricular block; *CABG*, coronary artery bypass grafting; *CAVB*, complete atrio-ventricular block; *COPD*, chronic obstructive pulmonary disease, *CRT-D*, cardiac resynchronization therapy with ICD capabilities; *DCM*, dilated cardiomyopathy; *ICD*, implantable cardioverter-defibrillator; *ICM*, ischemic cardiomyopathy; *LAFB*, left anterior fascicular block; *LBBB*, left bundle branch block; *LVEF*, left ventricular ejection fraction; *MRA*, mineralocorticoid receptor antagonist; *NICM*, non-ischemic cardiomyopathy; *PCI*, percutaneous coronary intervention; *RBBB*, right bundle branch block; *SGLT-2*, sodium-glucose cotransporter-2

### Procedural characteristics

Procedural characteristics of 34 patients in the ablation first group are shown in Table 2. The median time to VT ablation after the first VT presentation was 5 (IQR: 2–7) days. Subsequently, an ICD has implanted a median of 8 (IQR; 5–11) days after VT ablation. The median number of procedural VTs was 2 (IQR: 1–3), and the mean procedural time was 196 ± 56 min. With regards to the anatomical location of scar, the intraventricular septum was the site where most scars were identified (LV septal scar; 18/34 (53%), RV septal scar 6/34 (18%)). Acute success (complete abolishment) was achieved in 62% of patients, and partial success (elimination of at least 1 VT) in the remaining 38%. There were 3 (9%) procedural complications; specifically, these included the following: 1 episode of the anticipated atrioventricular block following CA of targeted clinical VT in the LV basal-septum, which was managed by biventricular ICD insertion; 1 episode of groin hematoma, which was treated with conservative management; and 1 episode of a small pericardial effusion (without any hemodynamic compromise, not requiring a pericardial drain or cardiac surgery). There was no intra-procedural death during the ablation procedure.

**Table 2:** Procedural characteristics in patients with early catheter ablation

	Ablation first (*n* = 34)
Time to VT ablation after initial presentation (days)	5 [2–7]
Procedure	
Number of VT inducible (*n*)	2 [1–3]
Procedural time (min)	196 ± 56
Approach	
Endocardial ablation (*n*, %)	34 (100)
Epicardial ablation (*n*, %)	1 (3)
Ablation strategy	
Pace mapping (*n*, %)	32 (94)
Activation mapping (*n*, %)	11 (32)
Scar homogenization (*n*, %)	19 (56)
Elimination of abnormal electrograms (*n*, %)	8 (24)
Anatomical location of scar	
Left ventricular	
Septum (*n*, %)	18 (53)
Anterior wall (*n*, %)	5 (15)
Inferior wall (*n*, %)	6 (18)
Lateral wall (*n*, %)3 (9)	
Right ventricular	
Septum (*n*, %)	6 (18)
Free wall (*n*, %)	4 (12)
Inferior wall (*n*, %)	2 (6)
Acute outcome	
Complete success (*n*, %)	21 (62)
Partial success (*n*, %)	13 (38)
Complications	
AV block (*n*, %)	1 (3)
Pericardial effusion (*n*, %)	1 (3)
Groin hematoma (*n*, %)	1 (3)

### Clinical outcomes

Class III AADs were discontinued in 5 out of 11 patients after the ablation procedure in the ablation group. As a result, 6 (18%) patients were on class III AADs post-ablation in the ablation first group and 65 (43%) patients in the ICD first group (ablation first group vs ICD first group, *P* = 0.006; Table 1). The median follow-up period was 625 (IQR: 274–1000) days post-ICD insertion. Of 184 patients, 127 patients (69%) were followed up for at least 1 year, and 80 patients (43%) were followed up for at least 2 years.

VA-free survival was higher in the ablation first, compared to ICD first groups (at median follow-up, 91% vs. 59%, log-rank test; *P* = 0.002; Fig. 1B). VA-free survival *off class III AADs* was higher in the ablation first, compared to the ICD first groups (at median follow-up, 71% vs 34%, *P* < 0.001). Survival free from the composite endpoints of VA recurrence, cardiovascular hospitalization, death, and transplant was also significantly higher in the ablation first group, compared to the ICD first group (at median follow-up, 84% vs 54%; log-rank test; *P* = 0.01; Fig. 1C).

### Clinical outcomes after excluding of VT storm patients

To verify the impact of VT storm for CA as a first-line therapy, we repeated the analysis after excluding VT storm patients. In this present study, storm presentation was observed in 20 patients (ablation first group vs ICD first group;12 (35%) vs 8 (5%), *P* = 0.002). As a result, 22 patients remained in the ablation first group and 142 patients in the ICD first group. VA-free survival was higher in the ablation first, compared to the ICD first groups (at median follow-up, 95% vs 61%; log-rank test; *P* = 0.02; Supplemental Fig. 1A). Survival free from the composite endpoint of VA recurrence, cardiovascular hospitalization, transplant, or death was higher in the ablation first, compared to the ICD first group (at median follow-up, 90% vs 55%, log-rank test; *P* = 0.01; Supplemental Fig. 1B).

#### Subgroup analysis depending on the underlying etiology

Furthermore, we performed the subgroup analysis depending on its underlying etiology, i.e., ICM and NICM. The treatment effect of early VT ablation for the primary endpoint (VA recurrence) was consistent across subgroups (ICM: log-rank test; *P* = 0.04, NICM: log-rank test; *P* = 0.02, respectively; Supplemental Fig. 2A, B). On the other hand, the treatment effect for the secondary endpoint (composite outcomes of VA recurrence, cardiovascular hospitalization, transplant, or death) was discrepant between subgroups (Supplemental Fig. 3A, B). Survival free from the secondary endpoint was higher in the ablation first, compared to the ICD first group only in the NICM subgroup (log-rank *P* = 0.02; Supplemental Fig. 3B). In contrast, survival free from secondary endpoints did not differ significantly between groups in the ICM subgroup (log-rank *P* = 0.23; Supplemental Fig. 3A).

### Predictors of VA recurrence

Univariate and multivariate logistic regression analyses were performed to investigate the independent predictors of VA recurrence after ICD insertion (Supplemental Table 1). A multivariate logistic regression analysis including gender, age, first-line CA, storm presentation, and LVEF as covariates revealed that first-line CA was the only independent protective predictor of VA recurrence, providing an 80% reduction in the risk of future VA recurrence, compared to the ICD first group (hazard ratio (HR) 0.20, 95% confidence interval (CI) 0.07–0.57, *P* = 0.003).

## Discussion

### Main study findings

This study compared the real-world clinical outcomes of a strategy of initial ablation with concurrent ICD implant with a strategy of initial medical therapy and ICD implant in patients with their first presentation of VT. Notably, 54% of the population had underlying NICM. The main findings of this study were as follows:

1. In patients with their first presentation of VT, an ablation-first strategy (with concurrent ICD implant post-ablation) was associated with significantly better outcomes compared to a strategy of initial medical therapy and concurrent ICD implant. The ablation first group had greater VA-free survival, greater survival free of a composite endpoint of VA recurrence, cardiovascular hospitalization, transplant, and death compared to patients treated with initial medical therapy and concurrent ICD implant.
2. The superior efficacy of the ablation first group in preventing VA recurrence was maintained regardless of underlying heart disease (ICM or NICM).
3. The ablation first strategy conferred an 80% risk reduction of VA recurrence in follow-up compared to the ICD first strategy after accounting clinical and procedural factors on multivariable analysis.
4. The ablation first strategy was safe with only 3% experiencing an unexpected complication and 9% experiencing any complication, with no intra-procedural deaths.

**Fig. 3:**
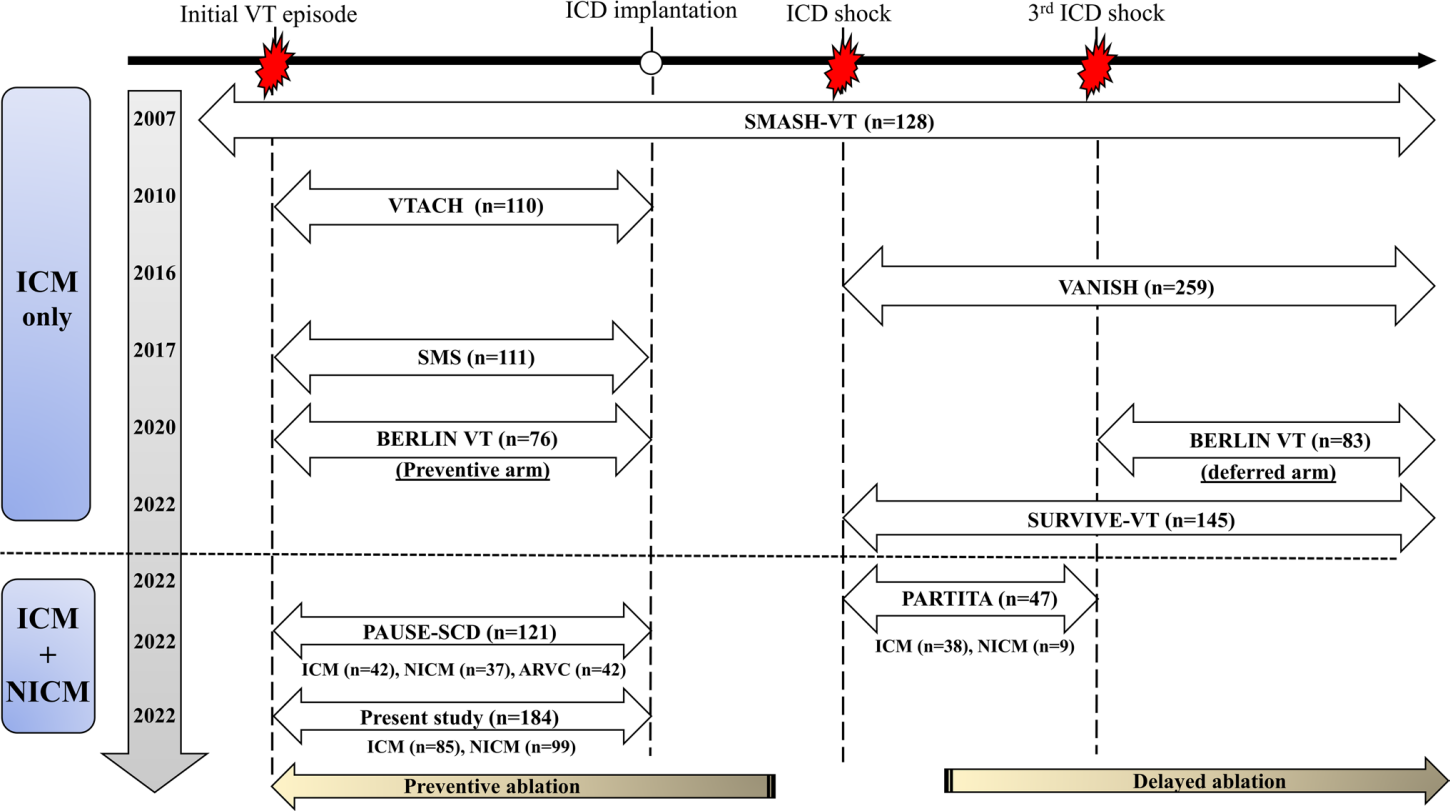
Schematic summary of prior randomized controlled trials

Despite contemporaneous randomized trial data that CA is superior to medical therapy for the treatment of VT [6–9, 22], in clinical practice, referral for VT ablation is generally deferred until multiple ICD activations have occurred and/or when aggressive Class I or III AAD therapy have failed to control VT. This approach often results in substantial patient morbidity, impaired quality of life, and recurrent health resource utilization from clinic visits and hospitalizations [4, 5, 23, 24] Notably, prior randomized studies of catheter ablation versus medical therapy for VT have been exclusively performed in patients with ICM [6–8, 13, 22, 25], with the exception of two recent trials PARTITA [9] and PAUSE-SCD [14] reporting a mixed population with 19% and 30% of NICM patients, respectively (Fig. 3). The present study, using real-world data, suggests that first-line CA (with adjunctive ICD implant thereafter) confers improved outcomes compared to the traditional approach of initial ICD implant and medical therapy in a mixed population with ICM and NICM. Our findings concur with those of PARTITA and PAUSE-SCD, strengthening the argument for earlier invasive intervention in VT, across all populations with structural heart disease.

### Prior studies

Several prior studies have examined the efficacy of catheter ablation, compared to medical therapy, at various time points of electrical instability in patients with initial presentation of VT (Fig. 3, Supplemental Table 2). Prior studies were summarized in the supplemental material.

Of particular note are two trials that included patients with NICM. The PARTITA trial, a prospective, multicenter, randomized clinical trial, has been performed to verify the prognostic effect of early VT ablation after the first ICD shock [9]. In this trial, patients with ICM or NICM and primary or secondary prevention indication for ICD who received the first shock for VT (*n* = 47) were randomly assigned 1:1 to VT ablation or continuation of standard therapy. Both “VT recurrence with ICD shocks” and a “combined endpoint of death or worsening heart failure hospitalization” were less frequent in the ablation group than in the control group (VT recurrence with ICD shocks; log-rank *P* = 0.039, combined or worsening HF hospitalization; log-rank *P* = 0.034), indicating the clinical benefit of early VT ablation.

The recent PAUSE-SCD assessed the efficacy of early, first-line VT ablation (before ICD implantation, which was earlier than in PARTITA) compared to conventional medical therapy in patients with cardiomyopathy of varied aetiologies. The results demonstrated that early, first-line VT ablation significantly reduced the composite outcome of VT recurrence, cardiovascular hospitalization, or death. These findings were driven by a reduction in ICD therapies.

Our results are consistent with both PARTITA and PAUSE-SCD trials, supporting the efficacy of early VT ablation. A simple comparison is not possible due to the different study designs; however, important differences exist between these two trials and our own study. First, in the PARTITA trial, VT ablation was performed after the first ICD shock, whereas in our study, VT ablation was performed earlier, after the first VT presentation prior to ICD insertion. Second, our population had a much higher proportion of patients with NICM (54%), compared to 19% in the PARTITA trial. Since NICM ablation has increased in recent years [17], our population is more in keeping with this trend, representing real-world data.

In PAUSE-SCD, 121 patients were included and randomized 1:1 fashion (early VT ablation vs conventional medical therapy; 60 vs 61). In comparison, our study is a retrospective study and includes a total of 184 patients. The notable point is that more than half of patients (55.4%) in PAUSE-SCD underwent an epicardial approach, whereas only one patient (3%) employed epicardial ablation in our cohort. Epicardial ablation might contribute to the improved outcomes for VT ablation [26]. Thereby, we may have underestimated the efficacy of upfront catheter ablation. However, at the same time, epicardial ablation may increase the risk of procedural complications. Even considering these differences, our study still shows that early VT ablation reduces 80% risk of VA recurrence in follow-up in mixed ICM and NICM cohort. This data indicates that early, first-line VT ablation is effective to reduce the VA recurrence in patients with cardiomyopathy of varied etiologies without necessarily performing an epicardial approach.

Finally, we performed the subgroup analysis depending on the underlying etiology. In both ICM and NICM subgroups, early VT ablation improved the survival-free from VA recurrence. However, early VT ablation did not reduce the cumulative incidence of the composite outcome in the ICM subgroup. The improvement of composite endpoint death and/or cardiovascular hospitalization by upfront CA is still controversial. SMASH-VT, VTACH, SMS, and BERLIN-VT failed to show the benefits of early VT ablation for the composite endpoint of ICD shock, death, and/or VT storm, whereas VANISH showed a favorable composite primary outcome. PAUSE-SCD also showed favorable composite primary outcomes of VT recurrence, cardiovascular hospitalization, or death, which was mainly driven by a reduction in ICD therapies. Our study showed the similar tendency of results for the efficacy of upfront CA with these previous studies. Early VT ablation shows the significant reduction of VA recurrence in both ICM and NICM subgroups; however, it did not show the clear benefit for the composite outcome from VA recurrence, cardiovascular hospitalization, transplant, and death, in patients with the ICM subgroup. Further studies will be needed to investigate the composite outcome including the mortality benefit of early VT ablation.

### First-line catheter ablation

One of the potential explanations for a better outcome with first-line CA is to stabilize the VT substrate in the early phase. In ICM, postinfarction remodeling starts developing just after the myocardial infarction [27]. These remodeling processes have been divided into 2 phases, an early phase (within 72 h) and a late phase (beyond 72 h), which are associated with time-dependent progressive dilatation and recruitment of border zone myocardium into the scar [28, 29]. Conducting channels tend to be present early postinfarction and are preserved over time [30]. Circuits that support VT early post-MI tend to be preserved over time [31]. Similarly, NICM is thought to be a progressive disease even under the optimal drug therapy [15], such as beta-blockers and ACE inhibitor/ARBs. These time-dependent progressive properties of the VT substrate might be related to the benefit of first-line CA that homogenize the VT substrate and block the VT circuits at the early phase.

As for iatrogenic complications, our data revealed that there were only 3 (9%) peri-procedural complications with no intra-procedural death, which is equivalent to the risk of ablation reported in larger cohorts of patients undergoing ventricular arrhythmia ablation and those reported in recent randomized trials [32, 33].

With the improvement of the mapping system and techniques, VT ablation is becoming a safer and more effective procedure. VT ablation was initially performed in the treatment of patients with frequent ICD shocks for VT, but it is now used more often and earlier in the management of VT. Our data supports the notion that first-line CA is safe and efficacious in patients presenting with initial VT. Further randomized controlled trials are needed to confirm our findings.

## Study limitations

This study had several limitations. First, this is a retrospective study from a single specialized center for VT management that performs a high volume of VT ablations, which creates the possibility of operator and selection bias that is unavoidable. Second is a small sample size, which might have been a hidden bias. Ideally, a large multicenter, randomized clinical trial would be conducted to minimize these biases and confounding factors. Third, inherently to a retrospective study, some patients were not followed-up for the duration of the median follow-up, especially in patients with ICD first group. This might limit the accuracy of the time-to-event analysis. Since this study was conducted in a large center, patients who had been stable for a certain period of time tended to be referred to the local clinics. However, to minimize the likelihood of underestimating recurrence, a referral system was in place with local clinics to report VA recurrence or other severe complications. Finally, our study takes no structured approach to AAD prescribing, but the benefit of this being the reflection of a real-world scenario.

## Conclusion

In this study, in which ICM and NICM population were equally represented, first-line CA at the time of the first VT presentation prior to ICD insertion results in significantly improved survival free of VA and survival free of a composite endpoint of VA recurrence cardiovascular hospitalization, transplant, or death, with a low incidence of peri-procedural complications. These real-world findings are consistent with recent randomized clinical trial data showing the benefit of early catheter ablation, compared to medical therapy at the time of the first presentation of VT.

### Supplementary Information

Below is the link to the electronic supplementary material.

Supplemental figure 1 (PNG 57 kb)

High resolution image (TIF 112 kb)

Supplemental figure 2 (PNG 54 kb)

High resolution image (TIF 109 kb)

Supplemental figure 3 (PNG 56 kb)

High resolution image (TIF 112 kb)

Supplemental tables 1 and 2 (DOCX 54 kb)

Supplemental material (DOCX 24 kb)

## Data Availability

The data that support the findings of this study are available from the corresponding author upon reasonable request.

## Abbreviations

AADs: Anti-arrhythmic drugs
ACE: Angiotensin-converting enzyme
ARB: Angiotensin receptor blocker
ARNI: Angiotensin receptor-neprilysin inhibitor
ATP: Anti-tachycardia pacing
CA: Catheter ablation
CMR: Cardiac magnetic resonance
ICD: Implantable cardioverter-defibrillator
ICM: Ischemic cardiomyopathy
IQR: Interquartile range
LAFB: Left anterior fascicular block defibrillator
LBBB: Left bundle branch block
MRA: Mineralocorticoid receptor antagonist
NICM: Non-ischemic cardiomyopathy
PES: Programmed electrical stimulation
RBBB: Right bundle branch block
SGLT2: Sodium-glucose cotransporter-2
VA: Ventricular arrhythmia
VT: Ventricular tachycardia
