# Supplementary figures and images for: Efficacy and safety of catheter ablation as first-line therapy for the management of ventricular tachycardia
Source: J Interv Card Electrophysiol. 2023 Feb 9;66(7):1701–11. doi: 10.1007/s10840-023-01483-2 (PMC10547804; doi:10.1007/s10840-023-01483-2)

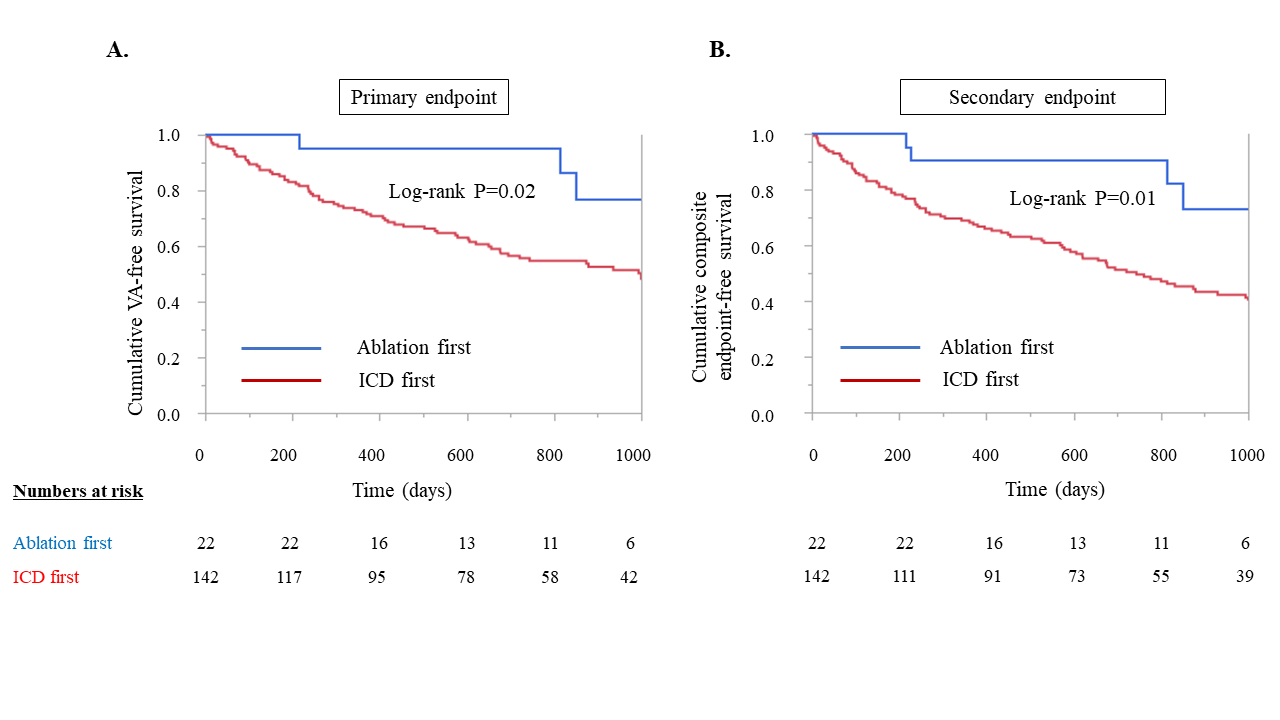

Supplement: Supplementary file 1 — (PNG 57 kb) [file 10840_2023_1483_Fig4_ESM.png]

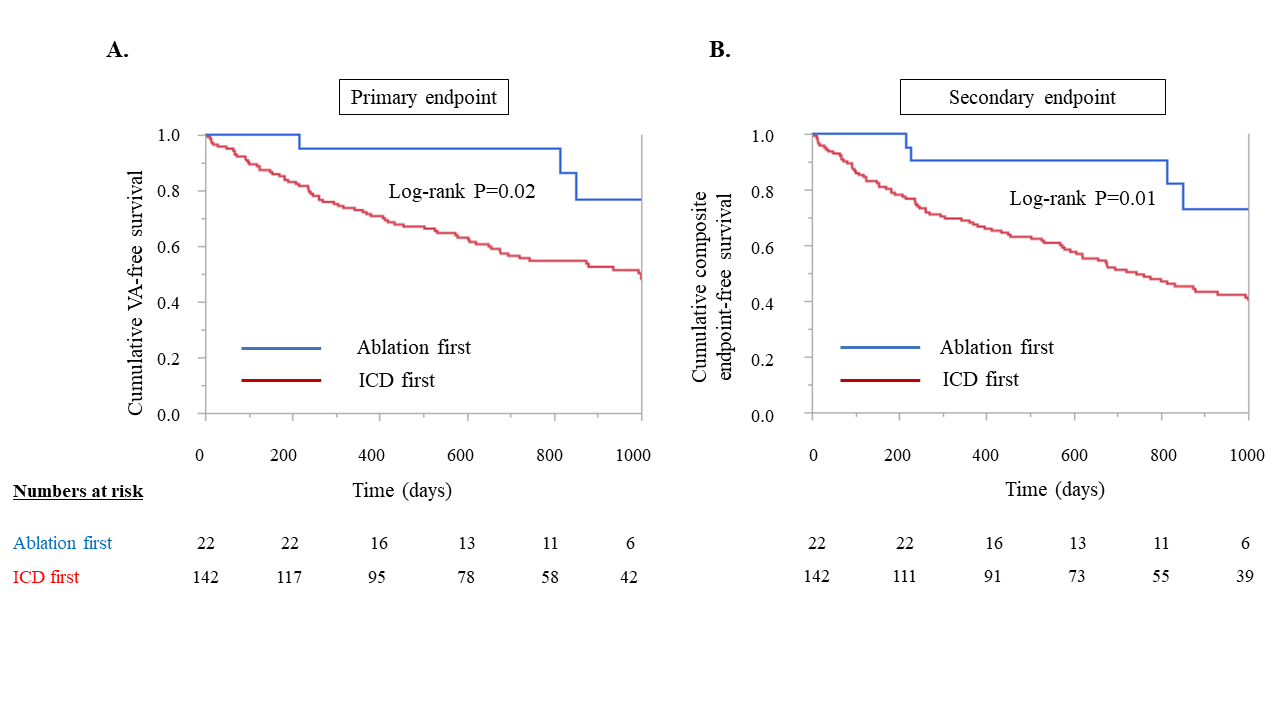

Supplement: Supplementary file 2 — High resolution image (TIF 112 kb) [file 10840_2023_1483_MOESM1_ESM.tif]

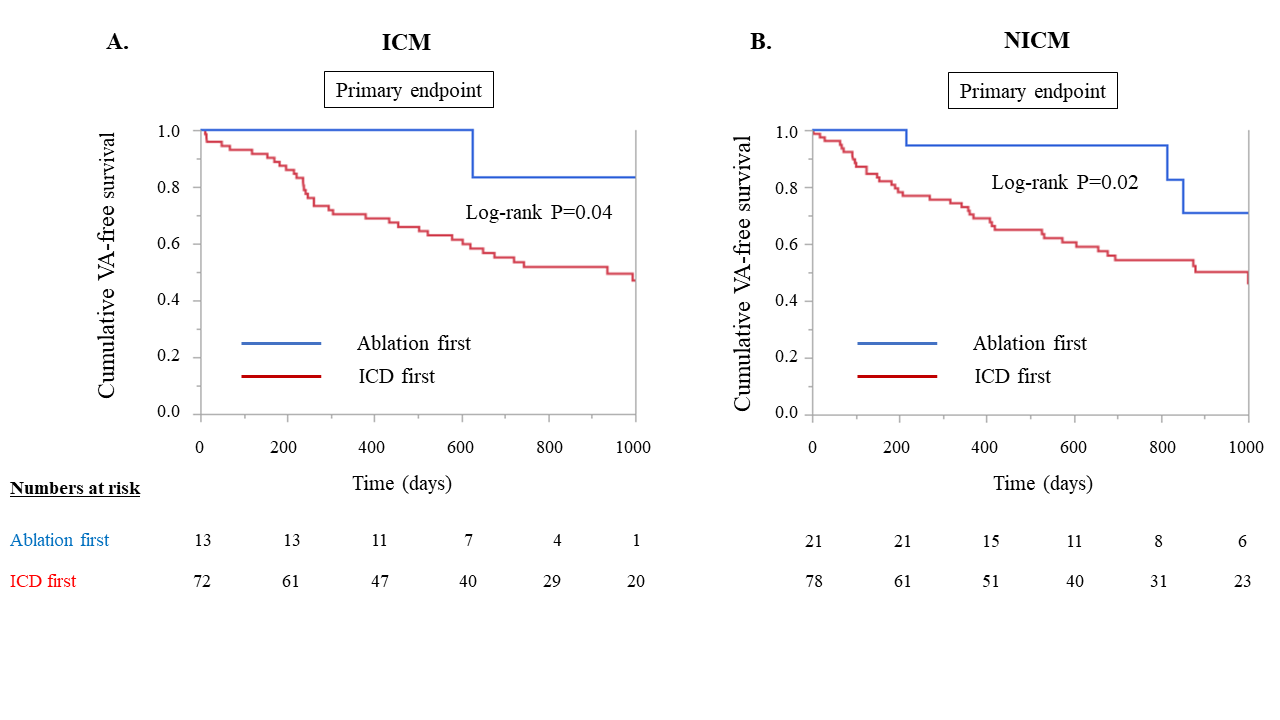

Supplement: Supplementary file 3 — (PNG 54 kb) [file 10840_2023_1483_Fig5_ESM.png]

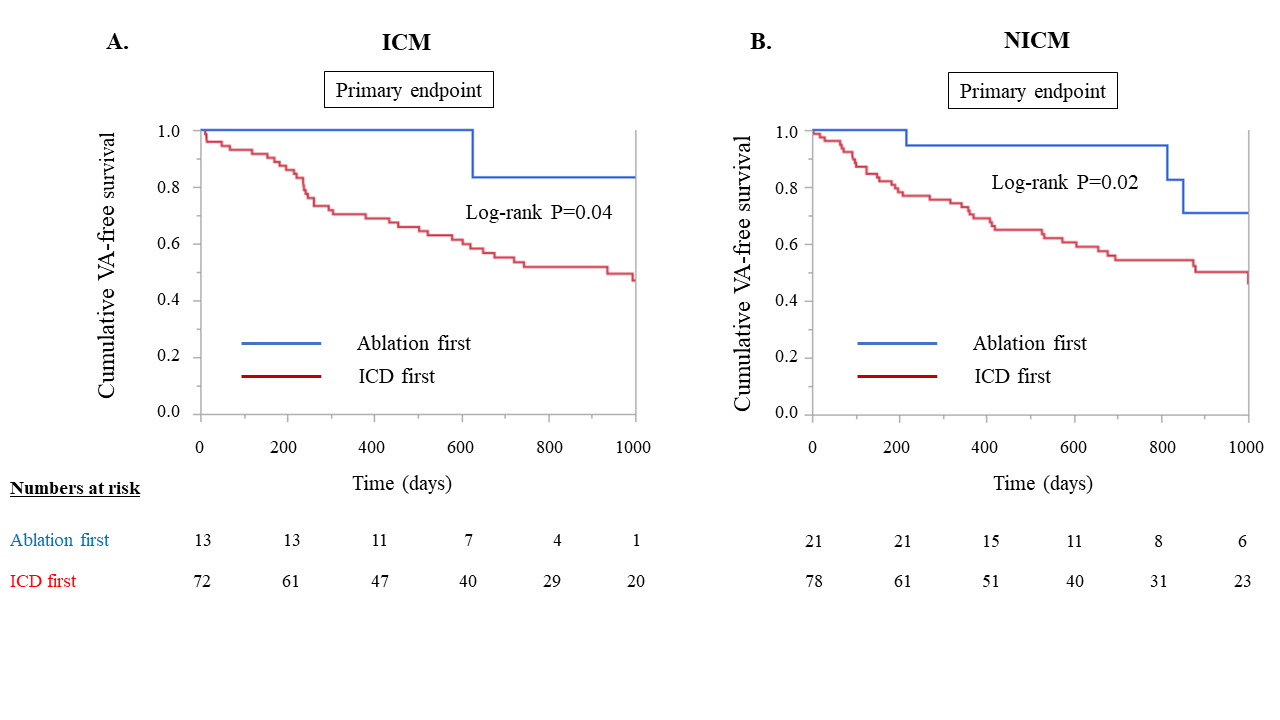

Supplement: Supplementary file 4 — High resolution image (TIF 109 kb) [file 10840_2023_1483_MOESM2_ESM.tif]

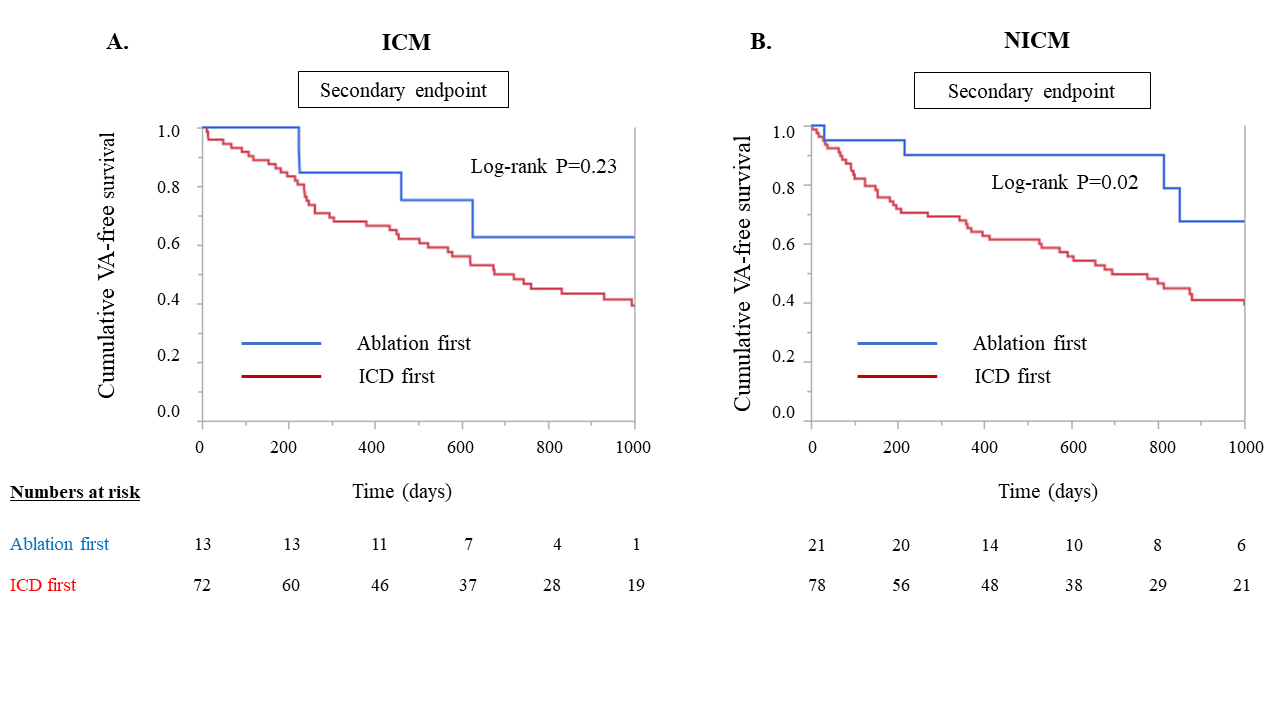

Supplement: Supplementary file 5 — (PNG 56 kb) [file 10840_2023_1483_Fig6_ESM.png]

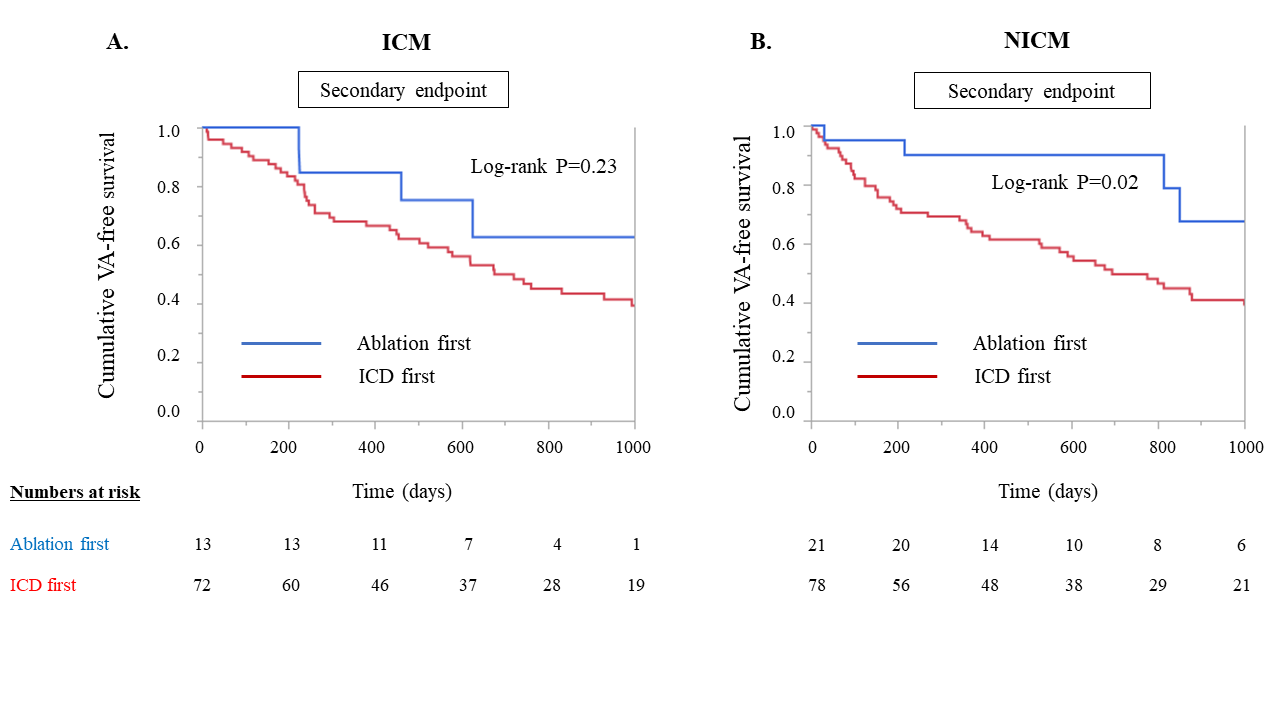

Supplement: Supplementary file 6 — High resolution image (TIF 112 kb) [file 10840_2023_1483_MOESM3_ESM.tif]
