# Supplementary material for: Efficacy and safety of catheter ablation as first-line therapy for the management of ventricular tachycardia
Source: J Interv Card Electrophysiol. 2023 Feb 9;66(7):1701–11. doi: 10.1007/s10840-023-01483-2 (PMC10547804; doi:10.1007/s10840-023-01483-2)
Supplement: Supplementary file 7 — (DOCX 54 kb) [file 10840_2023_1483_MOESM4_ESM.docx]

**Supplemental Table 1: Univariate and multivariate logistic regression analysis for VA recurrence after ICD insertion**

Variable Uni HR (95%CI) p value Multi (95%CI) p value

Male 1.39 (0.79-2.44) 0.25 1.26 (0.71-2.24) 0.43

Age > 65 1.04 (0.66-1.62) 0.88 1.03 (0.66-1.63) 0.89

First-line catheter ablation 0.24 (0.09-0.65) **0.005* 0.20 (0.07-0.57) 0.003***

Storm presentation 1.03 (0.47-2.25) 0.93 1.81 (0.79-4.14) 0.16

LVEF<35% 1.24 (0.76-2.04) 0.39 1.18 (0.72-1.96) 0.51

Ischemic aetiology 1.01 (0.64-1.58) 0.74

BMI >25 0.90 (0.52-1.54) 0.69

eGFR<60 1.05 (0.54-2.04) 0.89

Hypertension 1.22 (0.76-1.92) 0.39

Dyslipidemia 0.64 (0.41-1.00) 0.05

Diabetes mellitus 1.00 (0.62-1.62) 0.99

Atrial fibrillation 1.07 (0.67-1.69) 0.78

COPD 0.74 (0.30-1.83) 0.61

Previous PCI 0.83 (0.48-1.44) 0.50

Previous CABG 1.02 (0.57-1.82) 0.95

CRT-D 1.55 (0.92-2.61) 0.10

Supplemental Table 2

|  | Authors  (Year) | Comparison | Number of patients | Mean follow up | Results  of upfront ablation | Difference with present study |
| --- | --- | --- | --- | --- | --- | --- |
| SMASH-VT (2007) | Reddy, et al. (6) | “ICD + Ablation”  vs  “ICD alone” | 128 patients  (ICD + Ablation; 64, ICD only: 64)  All patients were ICM. | 22.5  months | Reduction of ICD therapy | - The exact time to ablation  from the first episode of  VT had not been reported.  - **Only ICM** patients with  **unstable** VTs were  included.  - Patients who received an  ICD for primary  prevention and had  received ICD therapy were  also included. |
| VTACH (2010) | Kuck, et al. (7) | “ICD + Ablation”  vs  “ICD alone” | 110 patients  (ICD + Ablation; 54, ICD only: 56)  All patients were ICM. | 22.5 months | Reduction of VA recurrence | - The exact time to ablation  from the first episode of  VT had not been reported.  - **Only ICM** patients with  **stable** VTs were included. |
| VANISH (2016) | Sapp, e t al. (25) | “ICD + ablation”  vs  “ICD + escalated AAD therapy” | 259 patients  (Ablation group; 132, escalated AAD therapy group 127)  All patients were ICM. | 27.9 months | Reduction of the composite of death, VT storm, appropriate ICD shock after a 30-day treatment period. | - VT ablation was  performed after the ICD  shock.  - **Only ICM** patients were  included. |
| SMS (2017) | Kuck, et al. (8) | “ICD + Ablation”  vs  “ICD alone” | 111 patients  (VT ablation + ICD; 54, ICD only: 57)  All patients were ICM. | 27.6 months | Reduction of the total number of ICD interventions.  VA recurrence rate did not differ between groups. | - The exact time to ablation  from the first episode of  VT had not been reported.  - **Only ICM** patients with  **unstable** VTs were  included. |
| BERLIN-VT (2020) | Willems, et al. (13) | “Preventive VT ablation”  vs  “Deferred VT ablation” | 163 patients  (Preventive ablation; 76, deferred ablation; 83, dropped out; 4)  All patients were ICM. | 14 months | Reduction of ICD therapy | - The exact time to ablation  from the first episode of  VT had not been reported.  - **Only ICM** patients were  included.  - Comparing patients with  preventive ablation with  deferred ablative  treatments. |
| SURVIVE-VT (2022) | Arenal, et al. (22) | “Catheter ablation (substrate-ablation)”  vs  “AAD” | 144 patients (Catheter ablation; 71, AAD; 73)  All patients were ICM. | 24 months | Reduction of the composite of death, appropriate ICD shock, hospitalization for worsening heart failure or sever treatment-related complications. | - VT ablation was  performed after the first  ICD shock.  - The exact time to ablation  from the first episode of  VT had not been reported.  - Only ICM patients were  included. |
| PARTITA  (2022) | Della Bella, et al. (9) | “Immediate VT ablation”  vs  “Continuation of standard therapy” | 47 patients (Immediate ablation; 23, standard therapy; 24)  Both patients with **ICM** and **NICM** were included. | 24.2 months | Reduction of VA recurrence  Reduction the risk of combined death or worsening HF hospitalization | - VT ablation was  performed after the first  ICD shock.  - Mostly patients included  in randomized trial (81%)  were **ICM.**  - Patients who received an  ICD for primary  prevention and had  received ICD therapy were  also included. |
| PAUSE-SCD  (2022) | Tung, et al. (14) | “First-line VT ablation”  vs  “Conventional medical therapy” | 121 patients (First-line ablation; 60, conventional medical therapy; 61)  Both patients with **ICM** and **NICM** were included. | 31.3 months | Reduction the risk of combined VA recurrence, death or cardiovascular hospitalization | - More than half (55.4%) of  patients underwent an  epicardial approach.  - Ablation-related  complications occurred in  8.3% of patients. |
