# Supplementary material for: Efficacy and safety of catheter ablation as first-line therapy for the management of ventricular tachycardia
Source: J Interv Card Electrophysiol. 2023 Feb 9;66(7):1701–11. doi: 10.1007/s10840-023-01483-2 (PMC10547804; doi:10.1007/s10840-023-01483-2)
Supplement: Supplementary file 8 — (DOCX 24 kb) [file 10840_2023_1483_MOESM5_ESM.docx]

Supplemental material

**Prior studies**

Several prior studies have examined the efficacy of catheter ablation, compared to medical therapy, at various timepoints of electrical instability in patients with initial presentation of VT (Figure 3, Supplemental Table 2). Studies that compared upfront ablation (with concurrent ICD), to medical therapy (and concurrent ICD implant) include SMASH-VT, VTACH, SMS, and the more recent PAUSE-SCD. These studies all showed that a strategy of initial ablation compared to medical therapy was superior in reducing VA recurrence and/or composite endpoints of VA recurrence, cardiovascular hospitalization or death (6-8,14). BERLIN-VT compared ablation after the first VT presentation (preventative ablation) compared to ablation after the 3rd ICD shock (deferred ablation) and showed preventive ablation was associated with a lower incidence of sustained VA and related ICD interventions although preventive ablation did not reduce the risk of composite endpoint of all-cause death and hospitalization for VA or worsening heart failure compared to a strategy of deferred VT ablation (13). VANISH, SURVIVE-VT and PARTITA compared catheter ablation versus medical therapy after one or more ICD shocks, showing risk reduction of the combined death or worsening heart failure hospitalization and/or ICD shocks endpoint with CA (22,25).
